# MedFit App, a Behavior-Changing, Theoretically Informed Mobile App for Patient Self-Management of Cardiovascular Disease: User-Centered Development

**DOI:** 10.2196/formative.9550

**Published:** 2018-04-27

**Authors:** Orlaith Duff, Deirdre Walsh, Shauna Malone, Lauri McDermott, Brona Furlong, Noel O'Connor, Kieran Moran, Catherine Woods

**Affiliations:** ^1^ Insight Centre for Data Analytics Dublin City University Dublin Ireland; ^2^ National University of Ireland Galway Ireland; ^3^ School of Health and Human Performance Dublin City University Dublin Ireland; ^4^ Department of Physical Education and Sport Sciences University of Limerick Limerick Ireland

**Keywords:** app development, cardiac rehabilitation, telemedicine, exercise, mHealth, focus groups, usability testing

## Abstract

**Background:**

The MedFit app is designed to facilitate participation of people with cardiovascular disease (CVD) in an exercise-based rehabilitation program remotely. This paper details the development for the MedFit app.

**Objective:**

The aim of this research was to develop a behavior change, theoretically informed exercise rehabilitation mobile app for adults with CVD by following the early stages of the formative research: development and feasibility testing.

**Methods:**

Adhering to the mobile health (mHealth) development evaluation framework, the stages of the formative research process including (1) development and (2) feasibility were undertaken. The content and format of the MedFit app were developed based on (1) theory, (2) usability testing, and (3) content design.

**Results:**

A systematic review of the literature was undertaken to identify the most appropriate theories from which to develop the app. This led to the creation of the MedFit app. The app went through iterative rounds of usability focus group testing with adults with CVD to provide feedback on the app. This process was framed by the unified theory of acceptance and use of technology model. Feedback was then translated into feasible technical improvements to be executed through close collaboration with the technical team, who adapted and made modifications to the app based on this codesign process.

**Conclusions:**

The formative research process of the app development involved theoretical underpinning, usability testing, and content design. mHealth interventions may play a key role in the future of health care, potentially addressing the barriers to participation in cardiac rehabilitation. This work will provide guidance for future research aiming to develop mobile apps by incorporating a best practice framework for mHealth intervention development and a user-centered design approach.

## Introduction

### Background

Cardiovascular disease (CVD) is the leading cause of mortality worldwide, accounting for 17.3 million deaths per year, which is expected to rise to more than 23.6 million by 2030 [1]. With the prevalence of CVD on the rise, secondary prevention methods to battle this condition have never been so important. Cardiac rehabilitation (CR) is a secondary prevention program. It is defined by the World Health Organization as the “sum of activity and interventions required to ensure the best possible physical, mental, and social conditions so that patients with chronic or post-acute cardiovascular disease may, by their own efforts, preserve or resume their proper place in society and lead an active life” [2]. CR involves exercise training, education on heart-healthy living, and counseling to reduce stress and help return to an active lifestyle. CR can be delivered within a hospital-based program and also via community-based programs to enhance long-term maintenance of CR participation. As physical activity (PA) has been shown to improve quality of life (QoL) and reduces mortality in patients with CVD, PA counseling and exercise training are the core components of the program. A Cochrane systematic review of exercise-based CR found that all-cause mortality was reduced by 26% (RR 0.74, 95% CI 0.63-0.87) [3]. CR has also been associated with reduced hospital admissions and improvements in psychological well-being and QoL [4].

Although the benefits of CR have been well documented, adherence to these programs is generally suboptimal. Across a number of surveyed countries, only 14% to 43% of cardiac patients participate in rehabilitation programs [5-8]. Poor uptake of CR has been attributed to several factors such as physicians’ reluctance to refer some patients, particularly women and people from ethnic minorities or lower socioeconomic classes, and a lack of resources and funding [9]. Furthermore, less than 50% of those who participate in CR maintain an exercise regime for as long as 6 months after completion of the program [10,11]. Results from a Cochrane systematic review revealed that common barriers to adherence to CR programs include accessibility and parking at local hospitals, a dislike of group environments, and work or domestic commitment [12]. This suggests that current CR programs do not suit all patients and that alternative modes of rehabilitation should be available. Mobile health (mHealth) technologies may hold the key to this new mode of CR delivery.

mHealth is a component of electronic health (eHealth) defined by the Global Observatory for eHealth as “medical and public practice supported by mobile devices, such as mobile phones, patient monitoring devices, personal digital assistants (PDA’s) and other wireless devices” [13]. According to Kailias and colleagues (2010), there are more than 7000 documented smartphone health apps available to the public [14]. mHealth technologies use techniques and advanced concepts from a multitude of disciplines such as computer science, electrical and biomedical engineering, health sciences, and medicine [15]. Technology-enabled health behavior change interventions are designed to engage people in health behaviors that prevent or manage disease [16]. mHealth may therefore address the previously cited poor uptake of CR and act as a useful tool in supporting the self-management of chronic disease [17,18]. Indeed, some of the core barriers as stated above (ie, accessibility, social unease, and difficulty engaging with CR because of work or domestic commitments) can be addressed through flexible mHealth solutions. The Institute of Medicine has even called to increase the design and testing of health technologies [19], with research into the effects and mechanisms of behavior change interventions also crucial [20]. mHealth solutions deliver many additional behavior change techniques (BCTs) that are not possible with standard pedometers, such as goal setting, social support, and cues to action [21]. These new techniques embedded within an mHealth framework may move toward helping to tackle one of the key issues of long term CR (ie, less than 50% of those who participate in CR maintain adequate levels of PA post 6 months).

Recent findings from Gallagher and colleagues [22] echo results from the Technology Usage Questionnaire [23] highlighting a high level of technology ownership or use within the CVD population. Previous research has found that most (77%) CVD patients indicated an interest in CR support through the Internet, 68% through the mobile phone, with many reporting interest in game-based CR (67 %) and virtual rehabilitation (58%) [23]. Therefore, mobile technology offers an important opportunity to improve access to secondary prevention for cardiac patients, particularly when modified to suit subgroups [22]. Advantages of mobile technologies for secondary prevention include access to psychoeducation at appropriate times, real-time tracking of behavior, and cues to action. Serious gaming designs can also be incorporated to highlight key healthy lifestyle behaviors across the lifespan [24,25]. Patients may also access health information and connect with health professionals and other cardiac patients more directly. Patients and health care professionals may benefit from a rich source of data, which can be in turn used to evaluate effectiveness. When mHealth avenues are incorporated or offered as an alternative to *traditional* CR (ie, hospital-based programs prearranged at set dates and times), improvements in multiple risk factors occur, and mortality benefits have shown to be equal for both modes of delivery [26].

Despite these potential benefits, it is extremely important to consider aspects of acceptance and engagement with mHealth interventions. This study adopts a multidisciplinary approach to development of the MedFit app, drawing on theories from engineering, computer science, and health psychology. For example, the development of the MedFit app has been underpinned by social cognitive theory [27] and the behavior change wheel (BCW) [28], as well as the unified theory of acceptance and use of technology (UTAUT) model [29]. These two models of health behavior change have been used to design how the best practice guidance and content will be delivered to the end user, whereas the UTAUT theoretical model aims to provide general determinants of technology acceptance, with previous research demonstrating how it can provide insight into key relevant predictors for technology acceptance [30].

It is vital to appropriately and adequately explore attitudes toward, as well as acceptance and usage of these devices [31]. However, there currently exists little research in relation to these emerging technologies and a community-based CR population who are aiming to maintain adequate recommended levels of PA in a long-term maintenance phase of CR. The aim of this study was to test usability and acceptance of the MedFit app and to test feasibility of app usage among the target CVD population. Multimedia Appendix 1 depicts the phases of intervention development and how the underpinning theory is related to the BCTs used, the focus group feedback, and feasibility field testing.

### Description of Alpha Medfit App (Preuser Testing)

MedFit is an mHealth app and is designed to allow people with CVD to participate in an exercise-based rehabilitation program remotely through an Android app. MedFit offers the potential to make exercise-based rehabilitation programs more effective by making them more accessible, more personalized, and more interactive by providing real-time support and feedback for participants.

The app comprised three central sections: exercise, progress, and my healthy lifestyle. Within the exercise section of the app, preset exercise programs were incorporated into the app. These programs consisted of a warm-up, main phase, and cool down, all of which can be performed in the comfort of the user’s own home. Local muscular endurance exercises as well as stretches were also incorporated into the programs. The dimensions of the exercise follow British Association for Cardiac Rehabilitation guidelines [32] for health-enhancing PA, including the minimum of 150 min of moderate intensity PA per week. Therefore, the general prescription for exercise will be based on the frequency, intensity, time, and type principle: frequency=variable (depending on time available to the patient), intensity=moderate or above, time=minimum of 150 min per week, and type=recommended aerobic exercises for CVD patient. These exercises are shown using exemplar videos that have been recorded by a qualified gym instructor.

The exercise section contained a *test yourself* function whereby users could do a 6-min walk test to test their progress. The *progress* section of the app contained user feedback displayed in charts and graphs so that the users could track their progress over time, for example, track step count. The *my healthy lifestyle* section of the app provided tips and recommendation on lifestyle factors such as healthy eating, alcohol consumption, PA, stress management, medication adherence, smoking cessation, and sexual functioning.

The app works in conjunction with a *Fitbit Charge HR* device and objectively measures PA and heart rate. Patients also received short message service notifications about their activity levels.

## Methods

### Overview of Methods

**Figure 1:**
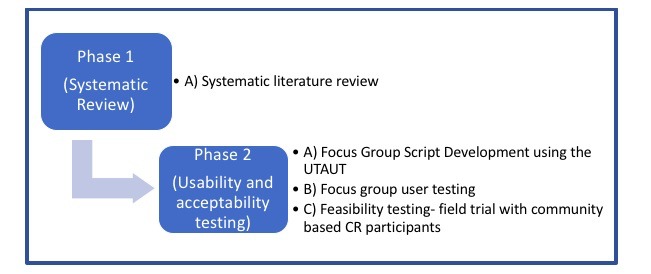
MedFit phased development process. UTAUT: unified theory of acceptance and use of technology; CR: cardiac rehabilitation.

This iterative development process encompassed two key phases, each with sub components. Phase 1 consisted of the systematic review and consultation with the advisory panel, whereas phase 2 involved usability and acceptability testing (using the UTAUT, focus group user testing and feasibility testing). See Figure 1 which depicts this process.

### Phase 1: Systematic Literature Review

The mHealth development and evaluation framework has been used to develop the app. The framework begins with the conceptualization phase. This phase in the MedFit apps development involved conducting a literature review. The MedFit research team conducted a systematic review [33] and identified what BCTs are used in PA eHealth interventions for people with CVD. The top three most frequently used BCTs included information about health consequences, goal setting (behavior), and joint third, self-monitoring of behavior and social support (practical). These BCTs were implemented within the MedFit app design to enhance user engagement and efficacy. From this review, the app content was designed and developed in line with the most frequently used groups of BCTs in the effective interventions. In tandem with this systematic review phase of the apps development, an advisory panel was established to review the proposed content emerging from the systematic review and to make recommendations. This advisory panel consisted of a multidisciplinary research team of experts in the areas of sport science, biomechanics, PA, electronic engineering, and health behavior change. Regular brainstorming sessions (ie, monthly) on how to best translate the theory and evidence into practical methods and techniques were held, whereby author OD generated content based on the current evidence base, and the advisory panel provided feedback before user testing within phase 2.

### Phase 2: Usability and Acceptability Testing of the MedFit App

#### Focus Group Script Development Using the Unified Theory of Acceptance and Use of Technology

To develop a theoretically informed focus group script, the UTAUT model was used [29]. The UTAUT 2 model was employed to ascertain the acceptance and use of mobile phone apps among MedEx Wellness participants. MedEx Wellness is a community-based exercise rehabilitation program for chronic illness located at Dublin City University. It offers supervised exercise classes to individuals with a range of chronic conditions, including CVD, pulmonary disease, diabetes, and cancer. A questionnaire (adapted from a questionnaire developed by Venkatesh et al (2012) [34]) entitled *Acceptability of mobile phone applications among adults with chronic illness* was completed by MedEx participants. A range of participants varying in age, sex, chronic condition, and duration of attendance at MedEx were recruited to the study.

The questionnaire comprised two sections (see Multimedia Appendix 2). Section 1 asked respondents about tablet computers and smartphones, asking if participants have either and whether they use mobile phone apps. Section 2 sought to obtain opinions regarding the importance of mobile apps using questions based on the UTAUT 2 model relating to participant opinions on factors such as *facilitating conditions, effort expectancy, social influence, performance expectancy,* and finally *hedonic motivation.* Respondents were asked to indicate the extent to which they agreed or disagreed with statements using a 7-point Likert scale response framework (1=strongly disagree, 2=disagree, 3=somewhat disagree, 4=neutral, 5=somewhat agree, 6=agree, and 7=strongly agree).

The role of the UTAUT2 questionnaire within this study was specifically to develop a theoretically informed focus group script that would pose questions relating to the core constructs identified as impacting on the acceptance and use of apps by participants. The focus group script also focused on the usability of the current prototype app.

#### Focus Groups User Testing

Participants in the focus groups were recruited from the HeartSmart program in MedEx Wellness that caters to individuals with CVD. In total, 26 HeartSmart participants took part in the focus groups (65% male; mean age=64 years, SD=8.2). There were five focus groups. Each focus group lasted approximately 1.5 to 2 hours in duration with a maximum of 6 people per group. The researcher aimed to balance the groups in terms of gender. The focus group was led by a moderator, who guided the interview, while an assistant moderator took notes on the ensuing discussion. The focus group had two main strands. The first focused on the usability of the MedFit app where the researcher presented the different functions of the app and the participants could follow along using a Samsung Galaxy S5 Neo on which the app was downloaded. Participants were asked to give their feedback and opinions on the prototype app components. The second strand of the focus group concentrated on the acceptability of the app with questions relating to the main constructs identified in the questionnaire that impacted participant’s acceptance and use of apps. The data were analyzed using content analysis [35].

#### Feasibility Testing—Field Trial With Community-Based Cardiac Rehabilitation Participants

A range of participants varying in age, sex, and duration of attendance at MedEx were recruited to the study (n=20; average age=69.4 years; range: 55-80 years). Three participants were unable to attend focus groups following the feasibility testing; therefore, this focus group is based on analysis of three groups consisting of 17 individuals. All participants were older than 18 years, had clinically manifested CVD, and were stable with regard to symptoms and pharmacotherapy for more than 4 weeks. Patients were excluded if they had cardiac disease or uncontrolled cardiac arrhythmias that limits exercise tolerance as identified by cardiac rehab staff, cognitive dysfunction that affects the consent process, severe joint pain that limits exercise tolerance, or had any of the American College of Sports Medicine exercise contraindications [36]. Participants then attended one session where they downloaded the app, set up an account, and were shown how to use the app. All patients were then given a user manual and helpline access. The MedFit app was given to each participant for a 2-week period. Following this 2-week period, participants were invited to a semistructured debrief focus group to provide feedback on the app. Full details of the debrief focus group script is available in Multimedia Appendix 3. This details the feedback that was sought from participants ranging from, for example, open-ended questions regarding app usage and experience to specific usability questions on each of the different components of the MedFit app. General feedback, as well as specific feedback on each of the components, was then sent to the technical team to update app further iterations.

Three focus groups were conducted which lasted approximately 1 to 1.5 hours in duration. There was a maximum of 7 per group.

### Data Analysis

#### Focus Group Script Development Using the Unified Theory of Acceptance and Use of Technology

To decipher what constructs played a role in participants use and acceptance of technology, the research team set a criteria whereby factors were rated positively if participants scored ≥15 on the 3-item constructs and ≥20 on the 4-item constructs on the positive end of the Likert scale; somewhat agree (5) or agree (6) or strongly agree (7).

#### Focus Groups User Testing

These focus groups were transcribed verbatim, while key notes were made on the usability section. Content analysis was used to analyze the data. Content analysis has several standard steps that were adhered to throughout the analysis [35]. First, an initial list was generated of ideas about the data and what was interesting about it with an initial set of codes generated for each focus group based on the data. This coding was done manually by going through the content of the entire dataset and linking the information to particular codes. From this step, a dataset was created whereby a full list of preliminary codes was available that emerged from the focus group data. Second, validation of this coding was undertaken whereby two members of the research team independently coded the same piece of transcription and then compared notes. Third, the preliminary codes were sorted into broader themes so that all the codes across each of the five focus groups belonging to a particular theme were grouped together. This stage was performed in Excel (Microsoft) whereby the researcher created a sheet for each focus group. Fourth, following this grouping of codes into potential themes, these themes were given separate columns, which included the relevant codes and illustrative participant quotes. Fifth, as one of the final steps in analysis, these preliminary themes were revised and refined. All the coded data extracts were reviewed to ensure they were appropriately coded to a given theme. The themes were then reviewed to ensure they accurately reflected the dataset and codes. The final sixth step involved defining and further refinement of the themes and subthemes [37].

#### Feasibility Testing—Field Trial With Community-Based Cardiac Rehabilitation Participants

These data were analyzed to identify both the general perceptions of the target group and the specific content, format, and navigation-related feedback. These perceptions and feedback were used to modify the relevant components of the intervention.

## Results

### Results From Focus Group Script Development Using the Unified Theory of Acceptance and Use of Technology

A total of 119 MedEx participants completed the UTAUT 2 questionnaire. Of these, 64.7% (77/119) of the respondents were male, with the average age of the group to be (n=116 [n=3 missing age data]) 65 years (SD 8.86; range=38-84 years). The duration of attendance in MedEx ranged from ≤1 month (15/119, 12.7%), 2 to 5 months (27/119, 22.9%), 6 to 12 months (18/119, 15.3%), 1 to 3 years (33/119, 27.1%), and >3 years (26/119, 21.8%). A total of 74.1% (88/119) of participants had a tablet computer, and 75.2% (90/119) owned a smartphone. A high percentage also revealed that they have used mobile apps on their smartphones (86/119, 72.3%).

Analysis of the UTAUT2 questionnaire revealed that performance expectancy, social influence, hedonic motivation, behavioral intention, effort expectancy, and facilitating conditions all rated highly among a majority of respondents. More than half of the respondents scored a total of 15 or more on performance expectancy, social influence, hedonic motivation, and behavioral intention (3-item constructs; see Textbox 1). Greater than half of the respondents scored a total 20 or more on the two 4-item constructs, effort expectancy and facilitating conditions. Almost three-fourths of the respondents from MedEx believed that they had the necessary conditions to facilitate the use of apps in their lives.

Only 22 (22/119, 18.9%) respondents scored ≥15 on the habit construct, indicating that end users did not perceive habit as playing a significant role in the acceptance and use of mobile apps among this cohort. A total of 40.2% (48/119) of respondents scored a total of 15 or more on the price value construct, indicating that perhaps price value does not play as significant a role as some of the other constructs. The results of the questionnaire were used to inform and develop the usability focus group script (Multimedia Appendix 3).

### Results From Focus Groups User Testing

Following in-depth content analysis, four main themes emerged. These were as follows: support, the app as a mentor or guide, translation of activity from gym to home, and technology knowledge gap.

See Multimedia Appendix 4, which provides a list of the feedback from the focus groups based on each app component and the translation of this feedback in app content.

#### Support

##### Learning or Familiarization Process

Participants placed huge emphasis on an initial familiarization and setup process. Many participants who weren’t familiar with using apps on a regular basis said that it would be very important to have a familiarization period where they would be taught how to use the app either in a one-to-one training session, *one-to-one would be great* (focus group 2), or in *small groups* (focus group 2). It was reiterated across the groups that learning how to use the app would occur over time using a *trial and error* method (focus group 1). However, at the initial introduction to the app, participants would need to be shown how to use the app in a simple step-by-step manner. One participant stated the following:

And it’s the lady bird approach. Right from the start, don’t assume any knowledge.

Focus group 3

Participants felt that they would also need written instructions or guide to help them learn how to use the app. This would also be helpful if they forgot how to use the app at home as they would something to look at for guidance:

Well a guide is always good...and that’s the only reason so if you don’t use something often you can come back to it without having to go miles to find out.

Focus group 5

These instructions or guide could also come in video format as this format will be familiar to them from CR:

...or even a video. I mean that’s what they use in cardiac rehab instead of doctors talking.

Focus group 5

**Themes from focus groups user testing.**

Themes

1. Support
  - Learning or familiarization process
  - Support from family or friends
  - Technical support
2. App as a mentor or guide
3. Translation of activity from gym to home
4. Technology knowledge gap

##### Family or Friends Support

Overall, most participants believed they would get support from family and friends to use the app. This support would come in the form of encouragement to use the app. Most people have families who are interested in their loved ones health and would therefore provide encouragement to use the app if they believed it would benefit their health, as illustrated in the following statement:

Most families, most people are lucky enough to have people interested in them. When you get sick, the first thing they do, if there’s anything they can do to help you get better. If it’s just to encourage you to exercise, they’d be all too happy to do it.

Focus group 1

There were differing views in the groups as to whether friends or family could provide technical support to use the app. Some believed their family, particularly their children, would have the knowledge and skills to help them use the app:

There’s a lot that we don’t understand we ask the kids about, you know, and they show us.

Focus group 1

One participant thought their family wouldn’t take an interest in the app; that they have their own apps and interests to worry about; however, their friends might because they are of a similar age and interest level.

##### Technical Support

In terms of technical support, most participants agreed that they would need a contact for technical support in case they had an issue that neither they nor their family or friends could solve. The participants provided numerous suggestions as to what format the technical support should come in. Some suggested the use of a comment box where you could leave a message on the app regarding your query either straight to the technical team or to other users of the app:

Probably the comment box is the best.

Focus group 4

Participants agreed that the best form of technical support would be the availability of contact number that participants would ring during set hours:

Well if you have your contact details there that if you are stuck, eh you can ring in.

Focus group 2

#### App as a Mentor or Guide

The theme *app as a mentor or guide* was present in all five focus groups. Participants believed the app would provide instruction and knowledge on how to exercise correctly:

I think it’ll be useful in my life because...I’ll go to the gym and I have this to do my warm-up...shows me what weights to do, you know,...Because when you go sometimes you just haven’t a clue and you’re kind of doing stuff and you could hurt yourself, you could overdo it, it’s perfect, you know exactly what you’re doing and...keeps you healthy.

Focus group 1

Feedback and monitoring on their progress while using the app was seen as important to the participants, as illustrated in the following statement:

It’s important to get feedback.

Focus group 5

Participants liked the idea of *keeping up on things as they’re happening* (focus group 4) and expressed an interest in monitoring their progress on the app:

It would be kinda interesting watching what you’re putting in and seeing the progress or the opposite.

Focus group 4

Participants also believed that the app would heighten awareness to exercise and provide motivation to exercise in the form of prompts or cues (eg, push notifications), as illustrated in the following statements:

Because, I mean first of all it would motivate you, and it would also give you correct information and guide you where you’re going.

Focus group 5

I think we sit down a lot more than we realise, we drive a lot more that we realise, you know, I personally speaking and I think it would be sort of a wakeup call to me anyway. To actually see it in black and white.

Focus group 4

The code *app as a tool* came under the theme *app as a mentor or guide* as participants thought the app has a job or unction to do and did not necessarily have to be fun, as illustrated in the following participant quotes:

It’s good to have something there to support you but for me, personally it doesn’t need to be fun. It just needs to do what it says on the box, as they say.

Focus group 1

No it’s a tool...It’s there to do a job.

Focus group 4

The app would also motivate their family members to exercise having seen their family member use the app. Participants could see the benefit the app would have to the health of their family not just themselves:

I think it would benefit my own family. I have two teenage daughters that do like to sit down a lot when they’re at home, so I think if they saw me using the app at home they’d probably, probably slag the hell out of me but they’d probably eventually come out and join in and do something, yano.

Focus group 2

Yeah. I would say the only thing to do would be to try and include the family, in the programme.

Focus group 4

#### Translation of Activity From Gym to Home

Overall, the majority of participants agreed that the app would create an option for people to exercise who are housebound or for those who for one reason or another can’t make it to a structured exercise class:

Well I bring Mary from Rush but I have my own business so sometimes I can’t come and if I can’t come well Mary would have her app on her phone and I’d have it myself where you’d get a few minutes in the day where you can exercise, as I said rather than just saying ah I can’t go today I’ll sit down and have a rest.

Focus group 2

I’m living in Skerries, it’s not a great job having to get in but if Bridget is gone off in the car well I have to take a bus so eh, well now that makes me think about it again, use that or a bus? I think that would come out first and I would find myself using it.

Focus group 3

Participants viewed the app as part of building a healthy lifestyle:

Like I’d see this as part of building up a healthy lifestyle.

Focus group 5

The app would work in conjunction with structured programs, allowing for flexibility and planning, providing no excuse not to exercise, as illustrated in the following statement:

It means I can do it at home and I don’t feel like I’m slacking off.

Focus group 1

With that said, participants thought the app could be used in tandem with the gym or structured exercise classes. For the days that they don’t go to the gym, the app could be used instead to build up their activity to meet the guidelines:

Yeah sure you can make the sessions here what happens if you don’t make the sessions here but you but you know you’ve a period in the day where you can exercise...now you know what you can do and even if you go into a gym you’re going to go in and do something without damaging yourself.

Focus group 1

I would use it in tandem with the gym. I’d be more inclined to try and keep up with the gym but where I couldn’t do the gym, I would do it so. I might find that I got to the gym twice and use this once.

Focus group 1

#### Technology Knowledge Gap

Participants acknowledged that there is a generation gap when it comes to technology. Participants came from a generation where there were no smartphones and were therefore new to the concept of smartphones and their use of them. In comparison, it was acknowledged that today’s youth are familiar with technology and have little difficulty using smartphones:

And I mean that stuff is all so easy to the younger generation, even the seven year old granddaughter can use the bloody phone better than I can.

Focus group 1

Well I think you see you have a generational problem, here like...You’re talking to people who weren’t brought up with smartphones and apps.

Focus group 3

One woman also pointed out that they are not part of the *throw away generation* (focus group 3). She described this as where the older generations are more cautions than young people in trying out new technology in fear that they make break it, whereas younger generations have no fear associated with technology. Older generations came from a time where there was limited use of technology in their working lives and therefore are not up to speed with current smartphone advances.

It was also said that there may be a *fear of the unknown* associated with the use of apps on smartphones, as smartphones weren’t available as they grew up, as illustrated in the following participant quotes:

I’m totally illiterate with this stuff, I just...no matter how many times I’m shown I can’t do it.

Focus group 1

No no, well I’m just saying that like, I’m just anxious about it.

Focus group 2

However it was also acknowledged by a participant that smartphones are part of life and have multiple purposes:

The smartphone is part of my life. I look at football and everything on it.

Focus group 5

### Results From Feasibility Testing—Field Trial With Community-Based Cardiac Rehabilitation Participants

Following this in-depth analysis of each component, it was evident that there were three main usability issues remaining that arose in the second phase of debrief focus groups. These themes mapped directly onto existing themes from previous focus groups but interestingly provided insights into what needed to be further refined in addition to preliminary work done in each area. These themes were as follows: (1) support, (2) technology or knowledge gap, and (3) app as a mentor or guide.

Emerging from the feasibility testing, the feedback for each identified theme was more nuanced. Although the user manual and frequently asked questions (FAQs) were perceived as useful, phone support was cited a crucial aspect of support:

The user manual was great. I would have been lost without it as you are given so much new information at the start.

Focus group 1

I would always need a phone number to call for help.

Focus group 1

The *technology or knowledge gap* remained an issue within the feasibility testing, and confidence to use technology was not present across all participants despite familiarization:

I had to call for help 4 times in the fortnight.

Focus group 1

I am reluctant to try new technology.

Focus group 2

Many participants felt that they would not be able to download an app themselves and that enhanced support with even more extensive familiarization was needed:

I would not know how to download an app so would need help or instructions to do that.

Focus group 1

A presentation or video showing all of the functions at the app at the start would be useful.

Focus group 3

Indeed, many users noted that it was difficult to formulate what the technical issues were making aspects of the FAQ section almost redundant. Participants felt that it was difficult to explain technical issues via phone. A suggestion was that a repository where you could send screenshots of error messages would be useful and cut down on time spent with technical support on the phone:

When I am having problems with the app, I find it hard to put into words what is wrong when I don’t really understand it. I would like to be able to send pictures of what is happening.

Focus group 2

In relation to the app as a mentor guide, most participants did engage with the app and enjoyed the exercise component. However, most participants did not find the healthy lifestyle section useful or engaging.

Many cited that their PA levels were raised as a result of the app use. Checking activity progress was seen as a useful feature to receive accurate feedback on progress:

I found the progress part very useful. I got a reality check when I seen what I was doing and thought I was more active than I am

Focus group 3

Participants also found that the app made exercise accessible in a more flexible way by the virtue of being able to access the resources at home, which minimized barriers to attendance:

It let me do the exercises at home which cut out the time travelling to the gym.

Focus group 3

The app also provided variety in the routine, as illustrated in the following statement:

I like having many different exercise options, both the classes and app, which are suitable for my condition. It gives me variety and I feel safe.

Focus group 2

However, some people were concerned that using the app did not facilitate direct social interaction:

The app is only missing the nice atmosphere in the MedEx classes [community-based exercise] where you can talk to people in a similar situation.

Focus group 2

## Discussion

### Principal Findings

To the best of our knowledge, no studies have developed an app using the factors of the UTAUT, as well as health psychology theories (in particular the BCW, which facilitates detailed intervention description), with a CR app and wearable sensors among a typical CVD population. The development of a mobile app for exercise rehabilitation for adults with CVD was carried out in line with the mHealth development evaluation framework [38]. This paper detailing the formative research process, development, and feasibility testing is in line with the Medical Research Council’s framework for complex intervention design [39].

The creation of eHealth technologies is often led by a technology-driven approach as opposed to the user-centered approach, which could have been adopted for this project given the multidisciplinary nature of the team. Studies have shown that the full potential of eHealth technology can only be exploited when developed by a multidisciplinary team who apply a human-centered approach codesign approach with the specific context of the technology’s use in mind [37,40]. The research team aimed to develop a theoretically informed app with potential cardiac patients at the heart of the design. This design process was undertaken by a multidisciplinary team of health psychologists, PA specialists, and technology specialists. The team used a novel approach to application development whereby health behavior change theory and the UTAUT2 model were used to guide app development, with the patient voice at the heart of the mobile apps development.

This human-centered approach was vital given results indicating severe difficulties emerging from focus groups and field testing in terms of the technology or knowledge gap. Gallagher and colleagues have noted similar issues in a parallel population [22]. They highlight how age is frequently perceived as a critical barrier to technology engagement. People who are currently in the age range of 50 to 70 years tend to have technology but may not have engaged with full features of a smartphone with app capabilities [41]. Meanwhile, people younger than 50 years have a heightened exposure to technology in their everyday lives, thus having the capability to use more complex features. In contrast, people aged 70 years and older generally use devices in a more passive way, such as using a mobile phone for voice calls and receiving texts [42,43]. This can be seen within our MedFit sample. Previous research has shown that older adults tend to rely on younger people in areas where they are less confident, such as for setup and problem solving [43]. The large discrepancies between generations in relation to technology use are likely to dilute within the coming years because of the pervasiveness of technology in our everyday lives [41].

In relation to the mechanisms of behavior change, it is important to use theory to inform intervention design and to specify the BCTs used [44]. It has been well documented that behavior change interventions are poorly described in accurate and sufficient detail for readers to truly understand, evaluate, and replicate the intervention reported [45]. It is also apparent that interventions based on behavior change theory are more effective than those lacking a theoretical basis [46,47]. Therefore, we aimed to describe in detail the active ingredients of our intervention along with each development phase of the app, so that the apps development was easy to understand, track, evaluate, and replicate for future research.

### Strengths and Limitations

An important strength of this study is the theoretical underpinning of the MedFit app. Interestingly, it has been recently noted that wearable electronic monitors and mobile apps still lack several important BCTs [21]. In particular, empirically proven techniques such as action planning and problem solving are often absent from such apps [48]. This is an interesting avenue to explore as the MedFit app has built in core BCTs based on a systematic review conducted associated with intervention effectiveness; however, action planning and problem solving are not a part of the MedFit app.

Individuals with CVD were recruited using a convenience sampling method, and the participants in this study were selected from a community-based chronic illness exercise rehabilitation program; this sample may be somewhat different from those that never attend a community-based exercise program. Despite iterative phases of user testing within this study, a long-term testing period is needed. This is planned within the next phase of MedFit development.

This is particularly important given the results that a majority of participants had user difficulties with the MedFit app whereby they were not proficient with mobile apps and felt challenged by the MedFit app format. This is indeed a consideration that needs to be addressed in the future evaluation of the MedFit app. Indeed, it may be necessary in future work to also record level of technology use before participating in the MedFit trial to ascertain where the difficulties are based (ie, technology capability issues vs lack of interest in the MedFit app for CR delivery). Furthermore, it would be useful for future debrief interviews following MedFit app usage to provide parallel quantitative details, as well as qualitative data, to provide a more comprehensive picture of the acceptability of each of the app components.

### Directions for Future Research

This study explored the usability and accessibility of the MedFit app. This study has allowed us to gain feedback on patients’ issues using the app and gain feedback on elements that are easy and difficult to use. All relevant information has been shared with the technical team to allow for any feasible and necessary changes. This is important for the development and future implementation of MedFit. In particular, as noted in the introduction, it is important to highlight how uptake and sustained engagement with CR programs is a key issue for this research area. This study has started to explore how using MedFit can eliminate some of the core barriers to uptake and maintenance (ie, elimination of travel time, cost, and social anxiety through access to remote CR via an app); however, it is clear that these potential solutions can only be adequately evaluated and addressed in a full-scale pilot of the MedFit app.

The next step is the pilot of the MedFit app. An updated version of the app will be trialed in a pilot study to assess the app in a hospital-based trial that will involve participants who have recently completed hospital-based CR and are moving into the maintenance of long-term PA within the community. This will involve participants engaging with the app for a minimum of 4 weeks. Assessments will be completed pre and post the using MedFit use, which will include the following measures: cardiorespiratory fitness, PA, accelerometer data and questionnaires investigating PA, smoking, stress, medication adherence, alcohol consumption, and well-being. Additionally, focus groups and process measures will be implemented for the intervention group in their assessment following the intervention to gain an insight into their use of MedFit.

### Conclusions

This paper details the development of a mobile intervention for CVD patients. The development work has been carried out in a systematic approach from theory to user testing and technical team design expertise. This paper highlights the importance of transparency when designing mHealth interventions using BCTs and theory, so that interventions are easily understood, evaluated, and reproduced. The researchers have also demonstrated a novel way to examine the usability and acceptability of a mobile app within a focus group setting to ensure long-term technology adoption and use.

MedFit is an example of a person-centered approach combining mHealth and CVD secondary prevention. Mobile technology offers an important opportunity to improve access to secondary prevention and enhance CR programs, particularly for technology-literate participants who may face barriers to attendance of on-site CR [22]. Overall, it is hoped that the MedFit app will encourage the adoption of the mobile app to improve health behaviors, in particular the PA levels of people with CVD.

## Appendix

Multimedia Appendix 1 Intervention development.

Multimedia Appendix 2 Unified Theory of Acceptance and Use of Technology (UTAUT) Questionnaire.

Multimedia Appendix 3 Unified Theory of Acceptance and Use of Technology (UTAUT) derived focus group script.

Multimedia Appendix 4 Iterative focus group feedback.

## Abbreviations

BCT: behavior change technique
BCW: behavior change wheel
CR: cardiac rehabilitation
CVD: cardiovascular disease
eHealth: electronic health
FAQ: frequently asked questions
mHealth: mobile health
PA: physical activity
QoL: quality of life
UTAUT: unified theory of acceptance and use of technology
