# Supplementary material for: MedFit App, a Behavior-Changing, Theoretically Informed Mobile App for Patient Self-Management of Cardiovascular Disease: User-Centered Development
Source: JMIR Form Res. 2018 Apr 27;2(1):e8. doi: 10.2196/formative.9550 (PMC6334713; doi:10.2196/formative.9550)
Supplement: Multimedia Appendix 1 [file formative_v2i1e8_app1.docx]

Multimedia Appendix 1: Development processes of the MedFit App

| Social Cognitive Theory | Behaviour change wheel- intervention Functions) | BCTs (code number and title of BCT) | Co-design feedback (focus group themes) | App Content developed as a result of feedback |
| --- | --- | --- | --- | --- |
| Knowledge | Education, Training, enablement | 4. Shaping Knowledge  4.1 Instruction on how to perform a behaviour  9. Comparison of outcomes  9.1 Credible source | Support: Technical Support,  Technology knowledge gap  App as a mentor/guide | - **Healthy lifestyle** – Tips and recommendation on healthy lifestyle components - **Exercise** – Video and teaching points used to guide participants through each exercise. - **Progress** – Feedback on activity level |
| Perceived self-efficacy | Education, Training, Modelling | 6. Comparison of behaviour  6.1 Demonstration of the behaviour  6.2 Social comparison  15. Self-belief  15.1 Verbal persuasion about capability  15.3 Focus on past success | App as a mentor/guide  Support: Technical, family and friends, and the learning and familiarisation process | - **Exercise -** Demos of exercises, tests and feedback on tests and activity performed - **Social Interaction** – Provide support to participants by encouraging social interaction through the ‘MedFit group’. |
| Outcome expectations | Education, training, persuasion, modelling. | 5. Natural Consequences  5.1 Information about health consequences  5.6 Information about emotional consequences | App as a mentor/guide | - **Healthy lifestyle** – Tips and recommendation on healthy lifestyle components - **Notifications** – to help initiate and maintain the behaviour change |
| Perceived facilitators/  impediments | Education,Training, Enablement, Environmental restructuring, persuasion | 3. Social support  3.1 Social support (un-specified)  3.2 Social support (practical)  3.3 Social support (emotional)  12. Antecedents  12.1 Restructuring the physical environment  12.2 Restructuring the social environment | Translation of activity from gym to home  Support: Technical, family and friends, and the learning and familiarisation process | - **Social interaction** - Provide support to participants by encouraging social interaction through the ‘MedFit group’. - **Contact us** – Technical support number and information - **Exercise** – Ability to exercise anywhere and at any time |
| Goals | Education, persuasion, training. | 1. Goal setting and Planning, 1.1 Goal setting (behaviour), 1.2 Problem solving  1.3 Goal setting (outcome), 1.4 Action planning , 1.5 Review behaviour goal (s), 1.6 Discrepancy between current behaviour and goal , 1.7 Review outcome goal (s), 2. Feedback and Monitoring, 2.2 Feedback on behaviour 2.3 Self-monitoring of behaviour, 2.4 Self-monitoring of outcome (s) of behaviour , 2.6 Biofeedback, 2.7 Feedback on outcome (s) of behaviour, 7. Associations, 7.1 Prompts/ cues ,8. Repetition and substitution, 8.2 Behaviour substitution, 8.3 Habit formation, 8.4 Habit Reversal, 8.6 Generalisation of a target behaviour, 8.7 Graded task | App as a mentor/guide | - **Progress** – Individual, personalised goal given to get participant. Results and feedback provided on activity. - **Notifications** – to provide encouragement and support to users to reach their PA goal - **Exercise** – Classes adapted based on persons ability and needs |
