# Supplementary material for: MedFit App, a Behavior-Changing, Theoretically Informed Mobile App for Patient Self-Management of Cardiovascular Disease: User-Centered Development
Source: JMIR Form Res. 2018 Apr 27;2(1):e8. doi: 10.2196/formative.9550 (PMC6334713; doi:10.2196/formative.9550)
Supplement: Multimedia Appendix 2 [file formative_v2i1e8_app2.pdf]

ACCEPTABILITY OF MOBILE PHONE APPLICATIONS AMONG ADULTS WITH CHRONIC ILLNESS

Questionnaire

Instructions:

1) Please answer ALL questions in ALL sections.
2) Completion of this form will take 5-10 minutes.
3) The contents of this questionnaire will be kept strictly confidential.

Demographic Profile:

- Name: ____
- Date of Birth: ______________________________________
- Gender (Please circle appropriately): Male/Female
- How long have you been attending MedEx? (Please tick one box only)

☐ 0-1 month ☐ 2-5 months ☐ 6months - 1 year ☐ 1-3 years ☐ 3+ years

Tablet Computer and Smartphone:

Q1. Do you have a tablet computer e.g. Apple iPad, Kindle etc.? (A tablet is a wireless, portable personal computer with a touch screen interface. A tablet is typically smaller than a notebook computer but larger than a smartphone.)

Please circle ONE answer only

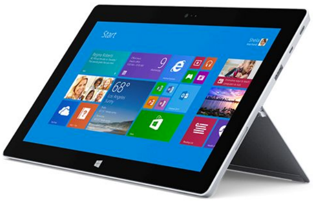

Yes No

Q2. Do you have a smartphone e.g., Samsung galaxy, iPhone etc.? (Smartphones allow you access the internet, apps, etc.)

Please circle ONE answer only

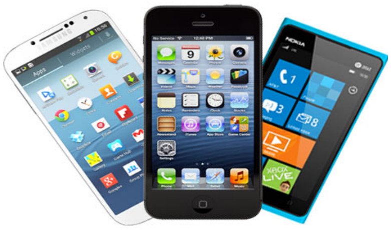

Yes No

Q3. If yes, is it an:

O Android phone
O iPhone (i.e. Apple iPhone)
O Other Smartphone: Please list ___________________________________

Q4. Do you use mobile applications (apps) on your smartphone e.g. Gmail, YouTube, Facebook? (A mobile app is a software application developed specifically for use on smartphones and tablets. To access an app you download it from an app store and click on the icon e.g. Gmail)

Please circle ONE answer only

Yes No

Section A: UTAUT 2

This section is seeking your opinion regarding the importance of mobile applications (apps) e.g. Skype, WhatsApp, Twitter. Respondents are asked to indicate the extent to which they agreed or disagreed to the following statements using the 7 Likert scale [(1) = strongly disagree; (2) =disagree; (3) = somewhat disagree; (4) = neutral; (5) = somewhat agree; (6) agree; (7) = strongly agree] response framework.

Please circle one number per line to indicate the extent to which you agree or disagree with the following statements.

No	Questions	Strongly Disagree	Disagree	Somewhat Disagree	Neutral	Somewhat Agree	Agree	Strongly Agree
**PE**	**Performance Expectancy**							
PE1	I would find mobile apps useful in my daily life.	1	2	3	4	5	6	7
PE2	Using mobile apps would help me to accomplish things more quickly.	1	2	3	4	5	6	7
PE3	Using mobile apps would increase my productivity.	1	2	3	4	5	6	7

No	Questions	Strongly Disagree	Disagree	Somewhat Disagree	Neutral	Somewhat Agree	Agree	Strongly Agree
**EE**	**Effort Expectancy**							
EE1	Learning how to use mobile apps would be easy for me.	1	2	3	4	5	6	7
EE2	My interaction with mobile apps would be clear & understandable.	1	2	3	4	5	6	7
EE3	I would find mobile apps easy to use.	1	2	3	4	5	6	7
EE4	It would be easy for me to become skillful at using mobile apps.	1	2	3	4	5	6	7

No	Questions	Strongly Disagree	Disagree	Somewhat Disagree	Neutral	Somewhat Agree	Agree	Strongly Agree
**SI**	**Social Influence**							
SI1	People who are important to me think that I should use mobile apps.	1	2	3	4	5	6	7
SI2	People who influence my behaviour think I should use mobile apps.	1	2	3	4	5	6	7
SI3	People whose opinions that I value prefer that I use mobile apps.	1	2	3	4	5	6	7

No	Questions	Strongly Disagree	Disagree	Somewhat Disagree	Neutral	Somewhat Agree	Agree	Strongly Agree
**FC**	**Facilitating Conditions**							
FC1	I would have the resources necessary to use mobile apps.	1	2	3	4	5	6	7
FC2	I would have the knowledge necessary to use mobile apps.	1	2	3	4	5	6	7
FC3	Mobile apps would be compatible with other technologies I use.	1	2	3	4	5	6	7
FC4	I would get help from others when I have difficulties using mobile apps.	1	2	3	4	5	6	7

No	Questions	Strongly Disagree	Disagree	Somewhat Disagree	Neutral	Somewhat Agree	Agree	Strongly Agree
**HM**	**Hedonic Motivation**							
HM1	Using mobile apps would be fun.	1	2	3	4	5	6	7
HM2	Using mobile apps would be enjoyable.	1	2	3	4	5	6	7
HM3	Using mobile apps would be very entertaining.	1	2	3	4	5	6	7

No	Questions	Strongly Disagree	Disagree	Somewhat Disagree	Neutral	Somewhat Agree	Agree	Strongly Agree
**PV**	**Price Value**							
PV1	Mobile apps are reasonably priced.	1	2	3	4	5	6	7
PV2	Mobile apps are good value for money.	1	2	3	4	5	6	7
PV3	At the current price, mobile apps provide a good value.	1	2	3	4	5	6	7

No	Questions	Strongly Disagree	Disagree	Somewhat Disagree	Neutral	Somewhat Agree	Agree	Strongly Agree
**HT**	**Habit**							
HT1	The use of mobile apps would become a habit for me.	1	2	3	4	5	6	7
HT2	I would become addicted to using mobile apps.	1	2	3	4	5	6	7
HT3	I must use mobile apps.	1	2	3	4	5	6	7

Section B: Behavioural Intention

This section is seeking your opinion regarding the importance of mobile applications (apps). Respondents are asked to indicate the extent to which they agree or disagree to the following statements using the 7 Likert scale [(1) = strongly disagree; (2) =disagree; (3) = somewhat disagree; (4) = neutral; (5) = somewhat agree; (6) agree; (7) = strongly agree] response framework.

Please circle one number per line to indicate the extent to which you agree or disagree with the following statements.

No	Questions	Strongly disagree	Disagree	Somewhat Disagree	Neutral	Somewhat Agree	Agree	Strongly Agree
**BI**	**Behavioural Intention**							
BI1	I intend to continue using mobile apps in the future.	1	2	3	4	5	6	7
BI2	I will always try to use mobile apps in my daily life.	1	2	3	4	5	6	7
BI3	I plan to continue to use mobile apps frequently.	1	2	3	4	5	6	7

- Would you be interested in participating in follow up focus groups? If yes, please provide a contact number.

O Yes   Contact Number: ___________________________________

O No

Thank you very much for taking part.

Should you have any further questions or if you would like to withdraw from the study, please do not hesitate to contact the researcher.

Orlaith Duff: 01-7007653
