# Supplementary material for: MedFit App, a Behavior-Changing, Theoretically Informed Mobile App for Patient Self-Management of Cardiovascular Disease: User-Centered Development
Source: JMIR Form Res. 2018 Apr 27;2(1):e8. doi: 10.2196/formative.9550 (PMC6334713; doi:10.2196/formative.9550)
Supplement: Multimedia Appendix 3 [file formative_v2i1e8_app3.pdf]

Focus Group Script

- Run with 5-6 people of mix gender and ages
- No more than 2 hours long with 10 minute a tea/coffee break in between
- Introductions (Hello and welcome, as you are aware this is a session to provide feedback on MedFit, a mobile app for cardiovascular rehabilitation)
- Explain how the focus group will work

Usability Section

<u>FitBit</u>

- Show participants the FitBit and its charger. Have the FitBit charged in advance so that they can see the different features of the FitBit.
- Ask participants to try the FitBit on to see what they think of it.
- Feedback screen – What would be the top three pieces of information shown on the screen e.g. heart rate, step count, flights of stairs climbed?

<u>App name:</u> Does anyone have any suggestions for the name of the app? MedFit is currently the demo name.

<u>App components</u>

Log in screen

- Show the participants the app login in screen.
- Are the visuals appealing and easy to interpret?
- Explain to participants that the initial setup will be on a laptop for security purposes and that they will then be given a login and password to access the app.
- Ask participants do they would find the process of typing in their login and password. Would it be easy? If not, what would be difficult about setting up an account?

Home page

- The home page includes the sections, exercise, progress and my healthy lifestyle. What do participants think of the home screen? Does it look too busy or is it laid out clearly?
- Can the participants decipher what is in each section before clicking into them? i.e. is the name of each section self-explanatory?

Exercise

- Explain the format of the exercise programme i.e. warm up, main phase, cool down and stretching and show the participants videos of how to do the exercises.
- Explain how each exercise is counted down i.e. 30 seconds
- Ask the participants - What do you think about this? What do you like? What do you not like? What would you change? Any other comments?
- Test yourself – explain the <u>6 minute walk test</u> and <u>sit to stand test</u>. (Don't clink into each – just explain that the test are similar to those completed in MedEx)*What do you think about this? What do you like? What do you not like? What would you change? Any other comments?*
- How do you think you would follow the exercises? Where would you place the phone? (Deirdre demo the exercises)

Progress

- Facilitator to show the participants feedback visualisation on the big screen to get feedback and goes through each piece of feedback on the dashboard systematically.

My Exercise Statistics

- My Activity bar chart (active mins per day): do you understand the information? Is there anything else you would like to see here? Any other comments? Anything you would change?
- Daily step count (progress bar indicates how many steps you have taken from 0 to 10,000): do you understand the information? Is there anything else you would like to see here? Any other comments? Anything you would change?
- Daily Heart Rate Information (average HR): do you understand the information? Is there anything else you would like to see here? Any other comments? Anything you would change?
- Weekly exercise goal: This is a prescribed goal which you can alter, which could potentially be based on step count and/or exercise sessions on the app. What do you think of this idea?
- Weekly exercise goal: In terms of the visuals on the screen, do you understand the information? Is there anything else you would like to see here? Any other comments? Anything you would change?
- Weekly workout time (hours and minutes): do you understand the information? Is there anything else you would like to see here? Any other comments? Anything you would change?
- Total exercise sessions: do you understand the information? Is there anything else you would like to see here? Any other comments? Anything you would change?

My Group's Exercise -> Found by clicking on the burger menu on the top right hand corner

- Group exercise duration: do you understand the information? Is there anything else you would like to see here? Any other comments? Anything you would change?
- Group attendance (sessions): do you understand the information? Is there anything else you would like to see here? Any other comments? Anything you would change?

Feedback Notifications: Go through handout

- Go through the examples of the rules
- What do you think of the idea of getting messages based on your progress/feedback and/or messages providing tips and recommendations during the week? Which would you be in favour of (i.e. progress or tips) and why? Would you like a combination on both types of messages? Any other comments?

My Healthy Lifestyle

- Show the screens for the health behaviours. Each section (e.g. physical activity) has recommendations and tips, as well as peer mentor videos and ask the expert videos. It could potentially have links to other relevant sources e.g. websites. What do you these ideas? Would you use this function? What do you like about it? What do you not like about it? What would you change about it?
- In terms of the peer mentor/ ask the expert videos: Would you watch them? What do you like about them? What do you not like about them? Would you use them as well as the text content? Instead of? Do they have any advantages/disadvantage above the text content?

Questionnaires

- Show the participants the example questionnaire on the iPad and how it will be filled in. What do you this of this? Do you think it would be easy/ difficult to answer the questions on a phone? Why or why not?

My MedFit Group

- Show the participants what is envisaged as part of this section i.e. events, message board, leader board.
- How would you expect to interact with other participants using mPATHway, if at all? What would you like about it? What would you not like about it?
- There will be an *events page* which will list local and national physical activity events. What do you think of this idea? Would you use this function? What do you like about it? What do you not like about it? What would you change about it?
- *Message board/chat function*? Explain briefly what we imagine the available social interaction features to be. Show an example of 'boards.ie'. What do you think of this

concept? Would you use this function? What do you like about it? What do you not like about it? What would you change about it?

- *Sample of Leader board* – Explain the concept of a leader board. Participants would be able to see the physical activity minutes/ step count of other users. Are there any other suggestions about what could be shown on a leader board?
- Would users be willing to have their name on a leader board or would you prefer to have an anonymous leader board with nicknames or I.D.'s for example?
- What do you think of the leader board? Would you use it? *What do you like? What do you not like? What would you change? Any other comments?*

Contact us

- Explain what is envisaged in this section e.g. video tutorials on how to navigate through the app and a section of frequently asked questions
- What do you think of this idea? Is there anything else you would like to see in this section? What do you like? What do you not like? What would you change? Any other comments?

Acceptance and use questions based on the UTAUT2

**Performance Expectancy**
1. Do you think you would find this app useful in your daily life? Why do you think that? If not, what do you think would make the app more useful?
2. Do you think this app would help you achieve the goals you set out in cardiac rehabilitation more efficiently? In what way do you think it will/will not help?
3. Do you think you would be more productive if you had this app to help you with your cardiac rehabilitation? Why do you think that?

**Effort Expectancy**
1. Do you think you would find learning to use this app easy? Why/ what parts of the application do you think make the app easy to use? If not, what could we do to make the app easier to use?
2. Do you think your interaction with this app would be clear and understandable? If not, what could we do to ensure that you could clearly understand and use the app?
3. In its current form do you think this app would be easy to use? If yes, what in particular makes it easy to use? If no, what suggestions/ feedback could you provide us with to make the app easier to use?
4. Do you think you could become skillful at using this app? Do you think it would take long to be able to understand and work the app properly yourself? Is there anything we could do that would help you to become skillful at using the app?

**Social Influence**
1. Do you think your family and friends would encourage you to use this app?
2. Why do you think they would encourage you to use the app? / Why would they not encourage you to use the app?
3. How could we make the app more appealing to your family and friends?
4. Is it important to you that your family/friends encourage you to use the app?

**Facilitating Conditions**
1. Do you think you have the resources necessary to use the app? E.g. money, time skill etc. If not, is there anything that could be done to facilitate easy use of the app?
2. Would you have the necessary knowledge to use the app? If not would you need detailed instructions on how to use the app e.g. instruction manual/ video tutorial?
3. Would the app be compatible with other technologies you use?

4. If you had difficulty using the app would you get help from family or friends? Do you think you would need IT support from our team in case you have a problem using the app? What form do you think this IT support should come in? (e.g. phone number for support) When should the IT support be available? (e.g. 9am-5pm Mon-Fri)

**Hedonic Motivation**
1. Do you think it would be fun/enjoyable/entertaining to use the app? Why/Why not? Is there anything that would make the app more enjoyable to use?

**Behavioural Intention**
1. Could you see yourself using the app regularly? Why/Why not?
2. Do you think you would try to use the app in your daily life? Is there anything that could be added/changed to make the app more appealing to use regularly?
