# Supplementary material for: MedFit App, a Behavior-Changing, Theoretically Informed Mobile App for Patient Self-Management of Cardiovascular Disease: User-Centered Development
Source: JMIR Form Res. 2018 Apr 27;2(1):e8. doi: 10.2196/formative.9550 (PMC6334713; doi:10.2196/formative.9550)
Supplement: Multimedia Appendix 4 [file formative_v2i1e8_app4.pdf]

Additional file 4: Participants usability feedback on the app

	Feedback on the App components	Actions taken
**Login**	➢ Need a password clue<br>➢ If the wrong password is inputted, have a link to retrieve password<br>➢ See letters come up on screen as you type your password	➢ Characters appear onscreen as person types in password<br>➢ Simple retrieve password function
**Home screen**	➢ Confusion regarding the 'burger' menu – many wouldn't know to click on it<br>➢ Change burger menu to the word 'menu'<br>➢ 'My healthy lifestyle' should not have the word my in it as this tab contains generic information<br>➢ Change 'My healthy lifestyle' icon	➢ Changed the 'burger menu' to the word 'menu'<br>➢ Removed the word 'My' from the title 'My healthy lifestyle'<br>➢ Changed the healthy lifestyle icon
**Exercise tab/exercise programme**	➢ Play video continuously under the timer<br>➢ Have a pause function in the exercise programme<br>➢ Play music with a beat. Option to mute the music<br>➢ Ability to log activity not picked up by FitBit.	➢ Video plays continuously under timer<br>➢ Ability to log activity not tracked by FitBit in new section called 'Log my activity'
**Progress**	➢ Need to see results/progress from the 'Test yourself' section<br>➢ Daily progress statistics should be the default screen<br>➢ Have range for the group attendance and duration but don't attach any personal identification - this would give people an idea of where they are in relation to the min and max<br>➢ Remove the group leaderboard	➢ Daily progress results are set as the default screen<br>➢ Removed identification from the group part of the app<br>➢ Removed the leaderboard
**Healthy Lifestyle**	➢ Happy with the information provided<br>➢ Use visuals to depict information<br>➢ Different levels of information – basic info, recent research, reference section to publications, links to additional sites for more information.	➢ More pictures used throughout the content<br>➢ Different levels of information provided to cater for all
**My MedFit group**	➢ Ability for users to add events to the event list or send them to the researchers via a comment box on the app<br>➢ Opt in/opt out function regarding the group chat function<br>➢ Potential to have a chat function/ comment box where users could message for tech support<br>➢ Small group chats (5-6 people)<br>➢ Remove the leaderboard	➢ MedFit group to be created in the version after the Beta version of the app. Feedback from the focus groups for this section of the app will then be incorporated into the app
**Menu**	➢ Video tutorial<br>➢ FAQ section<br>➢ Comment box<br>➢ Contact details for technical support<br>➢ Leave your details and a message and someone can get back to you (i.e. leave a comment)	➢ Video tutorial, contact details and FAQ all added to the menu function
**Feedback notifications**	➢ No more than 4 messages per week<br>➢ Suggestion to turn off notifications	➢ Maximum of 4 message sent per week

	FEEDBACK ON THE APP COMPONENTS	ACTIONS PLANNED
**LOGIN**	• Boxes for entering login in information too small	• Writing bigger<br>• App to remember username
**HOME SCREEN**	• Home screen is great. Buttons are very clear.	• No changes were required
**MENU**	• Many found this hard to see and hence did not access it.	• Changed word ‘menu’ to cog wheel icon – enlarged?
**EXERCISE CLASS**	• Many really liked exercise classes<br>• Would like a pause function for a few seconds before next exercise so the patient can see what is next.<br>• Would like to be able to pause and resume exercise classes	• Pause function implemented<br>• 5 second pause before each exercise implemented
**LOG ACTIVITY**	• Many did not see the role of this function	• The role is explained more in user manual
**EXERCISE TEST**	• Nice to be able to complete tests and see if you’re improving<br>• Many problems with internet	• Static screen implemented to state internet is required to complete this test.
**PROGRESS SCREEN**	• Really nice to see your progress<br>• Login can be annoying	• App to remember username
**HEALTHY LIFESTYLE**	• Writing for the education topics in the menu is too small.<br>• Information may need to be updated every few weeks – less generic	• Writing bigger in main tab
**FITBIT WRIST SENSOR**	• All really liked<br>• Irritated skin on 2 participants	
**USER MANUAL**	• Useful<br>• Some language too technical	• Language simplified
**FREQUENTLY ASKED QUESTIONS**	• Not useful	
**HELPLINE**	• Really useful – this would always be required<br>• Would like a contact us tab on app where screenshots can be sent to tech team – hard to explain issues	
**FEEDBACK NOTIFICATIONS**	• Too many<br>• Like content	• Will reduce number of messages sent
